# Development and Assessment of an Information Technology Intervention to Improve the Clarity of Radiologist Follow-up Recommendations

**DOI:** 10.1001/jamanetworkopen.2023.6178

**Published:** 2023-03-31

**Authors:** Jeffrey P. Guenette, Neena Kapoor, Ronilda Lacson, Elyse Lynch, Nooshin Abbasi, Sonali P. Desai, Sunil Eappen, Ramin Khorasani

**Affiliations:** 1 Center for Evidence-Based Imaging, Brigham and Women’s Hospital, Harvard Medical School, Boston, Massachusetts; 2 Department of Radiology, Brigham and Women’s Hospital, Harvard Medical School, Boston, Massachusetts; 3 Department of Medicine, Brigham and Women’s Hospital, Harvard Medical School, Boston, Massachusetts; 4 Department of Anesthesiology, Brigham and Women’s Hospital, Harvard Medical School, Boston, Massachusetts

## Abstract

**Importance:**

It is challenging to ensure timely performance of radiologist-recommended additional imaging when radiologist recommendation language is incomplete or ambiguous.

**Objective:**

To evaluate whether voluntary use of an information technology tool with forced structured entry of recommendation attributes was associated with improved completeness of recommendations for additional imaging over time.

**Design, Setting, and Participants:**

This cohort study of imaging report data was performed at an academic quaternary care center in Boston, Massachusetts, and included consecutive adults with radiology examinations performed from September 12 to 13, 2019 (taxonomy validation), October 14 to 17, 2019 (before intervention), April 5 to 7, 2021 (1 week after intervention), and April 4 to 7, 2022 (1 year after intervention), with reports containing recommendations for additional imaging. A radiologist scored the 3 groups (preintervention group, 1-week postintervention group, and 1-year postintervention group) of 336 consecutive radiology reports (n = 1008) with recommendations for additional imaging.

**Intervention:**

Final implementation on March 27, 2021, of a voluntary closed-loop communication tool embedded in radiologist clinical workflow that required structured entry of recommendation attributes.

**Main Outcomes and Measures:**

The a priori primary outcome was completeness of recommendations for additional imaging, defined in a taxonomy created by a multidisciplinary expert panel. To validate the taxonomy, 2 radiologists independently reviewed and scored language attributes as present or absent in 247 consecutive radiology reports containing recommendations for additional imaging. Agreement was assessed with Cohen κ. Recommendation completeness over time was compared with with 1-sided Fisher exact tests and significance set at *P* < .05.

**Results:**

Radiology-related information for consecutive radiology reports from the 4 time periods was collected from the radiology department data warehouse, which does not include data on patient demographic characteristics or other nonimaging patient medical information. The panel defined 5 recommendation language attributes: complete (contains imaging modality, time frame, and rationale), ambiguous (equivocal, vague language), conditional (qualifying language), multiplicity (multiple options), and alternate (language favoring a different examination to that ordered). Two radiologists had more than 90% agreement (κ > 0.8) for these attributes. Completeness with use of the tool increased more than 3-fold, from 14% (46 of 336) before the intervention to 46% (153 of 336) (*P* < .001) 1 year after intervention; completeness in the corresponding free-text report language increased from 14% (46 of 336) before the intervention to 25% (85 of 336) (*P* < .001) 1 year after the intervention.

**Conclusions and Relevance:**

This study suggests that supplementing free-text dictation with voluntary use of a structured entry tool was associated with improved completeness of radiologist recommendations for additional imaging as assessed by an internally validated taxonomy. Future research is needed to assess the association with timely performance of clinically necessary recommendations and diagnostic errors. The taxonomy can be used to evaluate and build interventions to modify radiologist reporting behaviors.

## Key Points

**Question:**

Was the voluntary use of an information technology tool with forced structured entry of relevant clinical attributes associated with improved completeness (including time frame, modality, and rationale) of radiologist recommendations for additional imaging?

**Findings:**

In this cohort study involving 1008 radiologist recommendations for additional imaging, implementation of a voluntary closed-loop communication tool that forces inclusion of time frame, modality, and rationale was associated with a significant 3-fold increase in complete recommendation content.

**Meaning:**

This study suggests that supplementing radiologist free-text dictation with voluntary use of a structured entry tool for clinical attributes was associated with increased recommendation completeness, which may enable improvement initiatives for timely performance of clinically necessary recommendations.

## Teaser

This cohort study evaluates whether voluntary use of an information technology tool with forced structured entry of recommendation attributes was associated with improved completeness of recommendations for additional imaging over time.

## Introduction

Radiologist recommendations for additional imaging are often subjective and variable.^1,2,3,4^ Between one-fourth and two-thirds of these recommendations are not followed.^5,6,7^ We could improve care quality and likely reduce diagnostic errors if we could ensure timely performance of clinically necessary recommendations for additional imaging and reduce clinically unnecessary recommendations for additional imaging. Two initial challenges emerge. First, reducing subjectivity and variability of recommendations for additional imaging requires modification of radiologist behavior. For example, we know that incomplete recommendations for additional imaging, such as those that do not include a recommended time frame, have a lower likelihood of being followed.^8^ Second, identifying and tracking recommendations for additional imaging is a challenge when radiologist language is incomplete or ambiguous.^9^

The American College of Radiology has launched an initiative to improve the communication and follow-through rates of these recommendations for additional imaging.^10^ Radiologist recommendation language can lack specificity and clarity.^8,11^ This lack of specificity and clarity is partly associated with radiologist diagnostic uncertainty.^12^ Attempts to follow the American College of Radiology initiative have focused on shifting radiologists toward more definitive reporting.^13,14,15^ Commonly accepted behavior theory suggests that we could increase effectiveness by also identifying and relieving diagnostic uncertainty and other forces that may be associated with radiologists’ variable and potentially ambiguous reporting tendencies.^16^

Several tools to track the completion of recommendations for additional imaging have been developed.^11,17,18^ These tools generally rely on consistent and comprehensive radiologist free-text reporting, yet we know that radiologist language is variable and potentially ambiguous.^8,11,12^ In 1 recent study of radiology reports, 62% of recommendations still did not include a time frame,^19^ making it challenging to assess the timeliness of recommendation completion. Furthermore, although validated taxonomies have been developed to analyze pathology reporting^20^ and medical error or incident reporting,^21,22^ for example, validated taxonomies have not been developed to categorize and analyze radiology reports. Taxonomies are designed to define and identify associated factors that may provide explanatory power,^21^ enable recognition of diversity and similarity,^21^ and enhance systematic organization learning.^22^ They are a mechanism for categorizing the narratives in reports.^22^ A taxonomy for recommendations for additional imaging would allow us to evaluate the completeness and ambiguity in the recommendations for additional imaging. This evaluation mechanism would enable validated and reproducible studies of the language of these recommendations and the effectiveness of tracking tools. It would also help identify radiologist- and examination-specific patterns in the completeness and ambiguity of the recommendations for additional imaging, allowing for the identification of the forces that underlie the incomplete and ambiguous language regarding these recommendations.

In this study, our primary aim was to evaluate whether voluntary use of a closed-loop communication tool that forces structured entry of recommendations for additional imaging was associated with improved overall completeness over time. Secondarily, we aimed to evaluate whether the communication tool occasioned completeness that was not provided in the associated free-text radiology report. To achieve these aims, we developed and internally validated a taxonomy to categorize and analyze the actionable vs confusing attributes of these recommendations for additional imaging.

## Methods

### Study Setting and Participants

This retrospective cohort study of imaging report data was conducted at a large academic quaternary care center. It was approved by the Mass General Brigham institutional review board with a waiver of informed consent due to the large number of retrospectively reviewed charts and deidentification of data prior to analysis and was performed in accordance with Health Insurance Portability and Accountability Act requirements. This report followed the Strengthening the Reporting of Observational Studies in Epidemiology (STROBE) reporting guideline for reporting observational cohort studies. Given the multiple concepts and components of this study, a study flow diagram with summary study results is included in the Figure.

**Figure.**
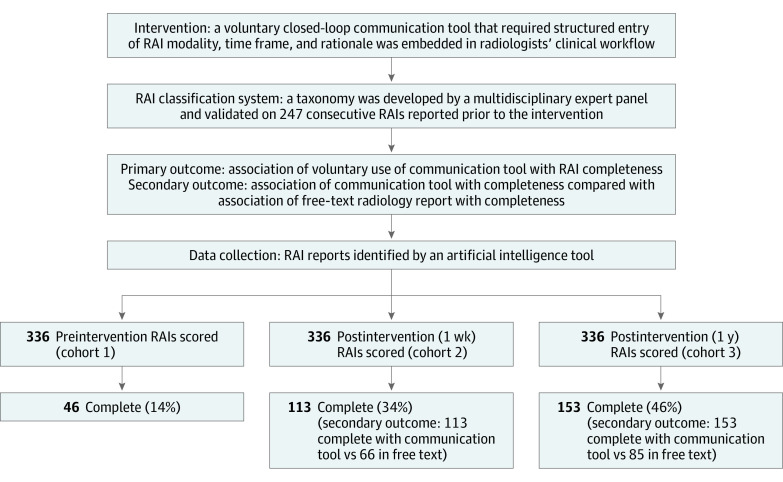
Study Flow Diagram With Summary Results RAIs indicates recommendations for additional imaging.

### Taxonomy Development

Based on feedback from a multidisciplinary expert panel of quality and safety leaders, the 4 radiology authors (J.P.G., N.K., R.L., and R.K.) iteratively constructed a taxonomy classification designed to identify the language choices for recommendations for additional imaging that cause confusion due to lack of specificity or clarity. Identified issue categories were unclear imaging modality, time frame, or rationale (not complete); equivocal, vague language (ambiguous); qualifying language (conditional); multiple options without delineation of the best option (multiplicity); and language dismissive of the ordered examination in favor of a different examination (alternate). Conversely, we thus considered the recommendations for additional imaging to be readily actionable if they were complete, unambiguous, and unconditional and without multiplicity or dismissive alternative language. As with other taxonomies, each category was designed to be independent from the others, and each category could be independently scored yes or no. A recommendation could be scored yes in all categories, no in all categories, or any combination of yes and no across categories. Additional details are provided in Table 1. Once this group of radiologists was in agreement on the taxonomy classifiers, the taxonomy was presented to hospital quality and safety leadership (S.P.D. and S.E.) for feedback, adjustment, and finalization by consensus.

**Table 1.** Radiologist Recommendation Taxonomy Definitions and Generic Examples

Taxonomy category	Definition	Example
Complete	Explicitly includes time frame, modality, and reason	Right frontal lobe 2-cm lesion, contrast-enhanced brain MRI is recommended within 2 wk for complete characterization
Ambiguous	Includes a general statement such as “continued follow-up” or “could be performed” or “as clinically indicated”	Right frontal lobe 2-cm lesion, contrast-enhanced brain MRI could be performed for further evaluation
Conditional	Contains an explicit conditional subordinate clause (typically an “if-then” or “when” statement) as part of an explicit recommendation	Right frontal lobe 2-cm lesion, if prior imaging cannot be obtained for comparison then contrast-enhanced brain MRI recommended within 2 wk for complete characterization
Alternate	Indicates or implies that a different examination would be more clinically useful than the examination being reported	Indeterminate right frontal lobe 2-cm lesion, MRI would be a better test
Multiplicity	Includes more than 1 modality for a single recommendation	Right frontal lobe 2-cm lesion, follow-up brain CT or MRI recommended

Abbreviations: CT, computed tomography; MRI, magnetic resonance imaging.

### Taxonomy Validation

The taxonomy was validated on a set of 247 consecutive radiology report impression sections that contained recommendations for additional imaging in examinations conducted September 12 and 13, 2019. This sample size was calculated based on prior unpublished data showing a detection rate as low as 3% for some of the taxonomy categories, a null of κ = 0.4 indicating that an agreement of less than 0.4 would be considered essentially random, and a target κ of 0.8 that would be considered excellent agreement.

Radiology reports containing recommendations for additional imaging were identified using an internally developed radiologist recommendation extraction algorithm based on BERT (Bidirectional Encoder Representations from Transformers), a deep learning framework introduced by Google in 2018,^23^ and the pretrained BERT_BASE_ model. Radiology reports from the following subspecialty divisions were included in each search: abdominal imaging, emergency radiology, musculoskeletal radiology, neuroradiology, noncardiac vascular imaging, nuclear medicine, thoracic radiology, and ultrasonography. Reports from other divisions (breast, cardiac, obstetrical ultrasonography) used unique reporting language and were therefore excluded. If different recommendations were provided for more than 1 finding in the same report, only the first recommendation was considered in this study for ease of data management, given the data were collected on a report-level basis.

Two board-certified diagnostic radiologists with 7 years (N.K.) and 3 years (J.P.G.) posttraining experience individually scored (yes or no) each of the taxonomy categories for the 247 recommendations for additional imaging. The interobserver agreement rate and the Cohen κ correlation coefficients were calculated for each taxonomy category in RStudio, version 2022.02.3 build 492 using R, version 4.2.1 (R Group for Statistical Computing), and the IRR package.

### Closed-Loop Communication Tool Intervention

A hospital-level quality improvement initiative called Addressing Radiologist Recommendations Collaboratively (ARRC)^9^ was developed as an expansion to a critical results closed-loop communication system^24,25,26^ to deliver clear radiologist recommendations for additional imaging and to track the acceptance and fulfillment of these recommendations. An early version of ARRC was trialed in our thoracic imaging group for the reporting of pulmonary nodules beginning October 2019.^11^

In brief, radiologists are highly encouraged to use the ARRC closed-loop communication tool (the ARRC tool) to communicate recommendations for additional imaging to referring clinicians. The ARRC tool interface requires the radiologist to specify a discrete time interval, imaging modality, and rationale for additional imaging (eFigure, A, in Supplement 1). The recommendation for additional imaging is then sent to the referring physician (eFigure, B, in Supplement 1) via an automated, ARRC-generated email notification, and the result is flagged in the clinician’s electronic medical record in-basket. The ARRC tool is separate from the radiologist dictation software. Thus, while it is requested that radiologists include a standardized sentence in the report noting that recommendations were filed in a closed-loop communication tool, the language in the report is otherwise left to the discretion of the radiologist. Division-level summaries were developed to show radiologist-level rates of recommendations for further imaging, including those filed with the ARRC tool and those not filed with the ARRC tool. These summaries include a list, by radiologist, of all report impressions containing a recommendation for additional imaging that was not filed with the ARRC tool. The summaries thus enable all radiologists in a division to see how their recommendation language and their use of the ARRC tool compare with their peers.

The ARRC initiative was announced and launched within the thoracic imaging division the week of October 21, 2019, and then gradually across other department divisions between April and December 2020. The first division-level reports were distributed between February 24 and March 27, 2021. Use of the ARRC tool was strongly encouraged but voluntary.

### Statistical Analysis

We used the taxonomy to assess whether the communication tool was associated with increased completeness of recommendations by scoring 3 sets of 336 consecutive radiology report impression sections that contained recommendations for additional imaging, for a total of 1008 scored impressions. The extraction process was the same as that used for the validation reports. The sample size was calculated for 1-sided Fisher exact tests with a significance threshold set at *P* < .05 and power set at 0.9 to detect a 25% between-group difference. We postulated that a minimum 25% change in behavior would constitute a considerable and likely meaningful change in behavior.

Analyzed recommendations for additional imaging were from radiology reports for examinations performed from October 14 to 17, 2019, the week prior to the initial implementation of the ARRC tool (time point 1); April 5 to 7, 2021, the week after the release of the first division-level summaries (time point 2); and April 4 to 7, 2022, 1 year later (time point 3). Information on whether these recommendations were filed with the ARRC tool was obtained from the ARRC database. A board-certified diagnostic radiologist with 3 years posttraining experience (J.P.G.) scored (yes or no) each of the taxonomy categories for the 3 sets of 336 recommendations for additional imaging.

Radiologists can generate a free-text recommendation that does not contain all of the complete attributes, yet they can file the recommendation in the ARRC tool, which forces inclusion of all the complete attributes of the recommendation. In other words, if a free-text recommendation lacks 1 or more of the complete attributes but the same recommendation is filed with the ARRC tool, it is by definition complete because of the ARRC tool forcing function. We therefore assessed both the change in completeness of free-text recommendations (complete free text) and the overall change in completeness, including recommendations with complete free-text language and/or complete via filing with the ARRC tool (complete overall).

To assess the primary outcome measure of whether the ARRC tool was associated with improved completeness of recommendations for additional imaging, 1-sided Fisher exact tests were performed to compare rates of reports with complete recommendations for additional imaging at time point 1 with time point 2 and to compare time point 1 with time point 3. Significance was set at *P* < .05.

In addition, a secondary outcome measure was the qualitative change in radiologist language associated with the ARRC tool. One-sided Fisher exact tests were performed independently for each taxonomy category (complete, ambiguous, conditional, alternate, and multiplicity), again comparing time point 1 with time point 2 and comparing time point 1 with time point 3. Mathematical correction was not made for multiple planned comparisons given these were exploratory secondary outcomes. An additional secondary outcome measure, whether the forcing function of the ARRC tool was associated with improved completeness of the recommendation compared with free-text recommendation language, was assessed with 1-sided Fisher exact tests and raw percentage differences at time point 2 and time point 3. Significance was set at *P* < .05. Statistical analyses were performed in RStudio, version 2022.02.3 build 492 using R, version 4.2.1.

## Results

### Patient Recruitment

Radiology-related information for consecutive radiology reports from the 4 time periods was collected from the radiology department data warehouse, which does not include data on patient demographic characteristics or other nonimaging patient medical information.

### Validation of the Taxonomy

The 2 radiologists had excellent agreement, with more than 90% agreement and a Cohen κ greater than 0.8 for all categories, thereby validating the taxonomy (complete: 96.8% agreement, κ = 0.87; ambiguous: 94.3% agreement, κ = 0.88; conditional: 98.0% agreement, κ = 0.94; alternate: 99.2% agreement, κ = 0.85; and multiplicity: 98.8% agreement, κ = 0.92). Selected illustrative examples of the application of the taxonomy are provided in Table 2. The taxonomy categories do not address diagnostic uncertainty, only the language expressed in the recommendation for additional imaging.

**Table 2.** Selected Illustrative Examples of the Taxonomy as Applied to Radiologist Recommendations for Additional Imaging in the Data Set for This Study

Recommendation	Complete	Ambiguous	Conditional	Alternate	Multiplicity
Patchy opacity in the right lower lung, likely pneumonia. Chest x-ray in 6-8 wk is recommended to assess for clearing.	Yes	No	No	No	No
Focus of asymmetric FDG uptake in what appears to be the right caudate in the brain. This is of uncertain etiology and may just represent asymmetric visualization of normal anatomy given slight head tilt. If the patient has signs or symptoms of neurologic deficits, a brain MRI could be performed for further evaluation.	No	Yes	Yes	No	No
Subcentimeter left upper lobe ground glass nodules, differential diagnoses include atypical adenomatous hyperplasia vs indolent adenocarcinoma spectrum lesions, less likely focal fibrosis. These may be reassessed on subsequent chest CT scan in 3-6 mo.	Yes	Yes	No	No	No
A single weight-bearing frontal view has been submitted; please note that this single view cannot assess the patellofemoral compartment or the presence of an effusion. A standard knee radiograph series can be obtained.	No	Yes	No	Yes	No
1.3-cm Intermediate attenuation lesion in the interpolar region of the left kidney. Likely a complex cyst but there are no priors to compare; recommend evaluation with ultrasonography or renal mass protocol MRI to further characterize	No	No	No	No	Yes

Abbreviations: CT, computed tomography; FDG, fluorodeoxyglucose; MRI, magnetic resonance imaging.

### Recommendation Completeness Using the ARRC Tool

The percentage of recommendations for additional imaging categorized as complete overall increased significantly from 14% (46 of 336) before implementation to 34% (113 of 336) 1 week after implementation (*P* < .001) and 46% (153 of 336) 1 year after implementation (*P* < .001).

### Change in Language Using the ARRC Tool

The percentage of recommendations for additional imaging with free-text language categorized as complete increased significantly from 14% (46 of 336) before implementation to 20% (66 of 336) 1 week after implementation (46 of 336 vs 66 of 336; *P* = .048) and 25% (85 of 336) 1 year after implementation (46 of 336 vs 85 of 336; *P* < .001). The percentage of recommendations for additional imaging categorized each as ambiguous, conditional, alternate, and multiplicity did not change significantly. However, there appeared to be a downward trend in the latter 3 categories (Table 3).

**Table 3.** Raw Numbers of the 336 Total Radiologist Recommendations for Additional Imaging for Each Time Frame Scored in Each Taxonomy Category With Fisher Exact Test for Comparisons With the Pre-ARRC Baseline

Time frame	Recommendations, No. (%) (N = 336)					
	Complete (overall)	Complete (report only)	Ambiguous	Conditional	Alternate	Multiplicity
Before ARRC initiative	46 (14)	46 (14)	198 (59)	67 (20)	12 (4)	18 (5)
1 Week afer ARRC initiative	113 (34)	66 (20)	214 (64)	65 (19)	6 (2)	26 (8)
*P* value	<.001^a^	.05^a^	.24	.92	.23	.28
1 Year after ARRC initiative	153 (46)	85 (25)	184 (55)	55 (16)	6 (2)	30 (9)
*P* value	<.001^a^	<.001^a^	.31	.27	.23	.10

Abbreviation: ARRC, Addressing Radiologist Recommendations Collaboratively.

^a^ Statistically significant.

### ARRC Tool Forcing Function

Use of the tool increased the completeness rate compared with free-text report language 1 week after the intervention (113 vs 66; *P* < .001) and 1 year after the intervention (153 vs 85; *P* < .001). Of the 113 complete recommendations for additional imaging in the 1-week postimplementation period, 87 (77%) were filed in the ARRC tool. Only 40 of the 87 recommendations for additional imaging (46%) contained complete language in the associated free-text report. Of the 153 complete recommendations for additional imaging in the 1-year postimplementation period, 124 (81%) were filed in the ARRC tool. Only 56 of the 124 recommendations for additional imaging (45%) contained complete language in the associated free-text report. In both periods, ambiguous, conditional, and multiplicity was present in the free-text reports associated with recommendations for additional imaging that were filed with the ARRC tool, including ambiguous language in 32 of 87 of these free-text reports (37%). Results are presented in Table 4.

**Table 4.** Raw Number of Radiologist Follow-up Recommendations Scored in Each Taxonomy Category for Reports Filed in the ARRC Tool^a^

Time frame	ARRC, No.	Recommendations, No. (%)				
		Complete	Ambiguous	Conditional	Alternate	Multiplicity
1 Week after ARRC initiative	87	40 (46)	28 (32)	2 (2)	0	5 (6)
1 Year after ARRC initiative	124	56 (45)	36 (29)	10 (8)	0	12 (10)

Abbreviation: ARRC, Addressing Radiologist Recommendations Collaboratively.

^a^ These recommendations were ultimately all complete, even if the complete information was not included in the report, due to the forcing function in ARRC.

## Discussion

To evaluate whether voluntary use of an information technology tool that forced structured entry of radiologist recommendations for additional imaging was associated with improved overall completeness of recommendations for additional imaging compared with free-text report language, we developed and internally validated a taxonomy. Using the taxonomy, we confirmed that implementation of a closed-loop communication tool (the ARRC tool) was associated with a significant increase in completeness of recommendations for additional imaging (defined as including time frame, modality, and reason) from 14% of recommendations before implementation to 46% of recommendations 1 year after implementation. Furthermore, the taxonomy clarified that the forcing function within the ARRC tool increased the number of complete recommendations for additional imaging relative to the associated free-text reports. However, nearly one-third of the complete recommendations filed with the ARRC tool still contained ambiguous language in the associated free-text radiologist reports.

Our approach to building this taxonomy was based on a taxonomy designed for categorizing and evaluating pathology reports^20^ and on a taxonomy designed for categorizing and evaluating family medicine error reports.^21^ The concept of using a taxonomy to categorize and analyze recommendations for additional imaging has been recently explored.^19^ That taxonomy was not validated, and the taxonomy presented here is less complex because it does not contain multiple domains. In this study, we applied our taxonomy to track the association between an intervention and the recommendations for additional imaging. However, the taxonomy would also be suitable for exploring what situations are associated with unclear recommendations (and then evaluating interventions to address those situations). The taxonomy would also be suitable for identifying specific individuals who frequently provide unclear recommendations (and to track the outcome of interventions aimed at those individuals).

In this study, we found that the free-text language of these recommendations often lacks completeness even when the radiologist has simultaneously filed a recommendation with the ARRC tool, which forces the radiologist to include the recommended examination modality, time frame, and reason. The free-text dictation and the ARRC closed-loop communication tool exist separately, and therefore the dictation language and the recommendation filing in the tool are both subject to radiologist discretion. This finding demonstrated that free-text radiology reports cannot be relied on to contain adequate information for optimal patient care without some type of function that forces completeness (explicitly including time frame, modality, and reason). As such, natural language processing and artificial intelligence tools that search free-text reports are unlikely to be fully effective in identifying and tracking appropriate recommendation completion or fulfillment, unless the reports are autopopulated by a tool with a specificity forcing function.

It has been previously shown that explicitly mentioning the recommended interval as part of a recommendation for additional imaging is associated with a 3-times-higher likelihood of the recommended additional examination being performed.^8^ We postulate that the rate of fulfillment of recommendations for additional imaging will therefore be higher for complete recommendations as well as unambiguous recommendations than for incomplete and ambiguous recommendations. Further research will be needed to assess whether the fulfillment rate does truly increase, either due to increased complete reporting or due to reduction in overall recommendations due to decreased frequency of recommending additional imaging when the recommendation would have included ambiguous language. Further research will also be needed to evaluate whether a higher resulting fulfillment rate will be beneficial for patients. Conditional recommendations may have value in certain circumstances, but we suspect that they are likely overused. Recommendations that contain alternate language are challenging because they imply that the ordering clinician ordered the wrong test. Recommendations with a multiplicity of choices defer the choice of imaging examination to another clinician, who may not know the pros and cons of the imaging examination options, and making it difficult to monitor the fulfillment and appropriateness of the recommendation.^9^

With varied use of language, the radiologist deflects the decision on when and whether to obtain more imaging back to the ordering clinician. In our discussions, ordering clinicians interpret this behavior in various ways, including feeling frustrated due to perceived ambiguity. Similarly, while we know that radiologist recommendations have a clinically important yield in diagnosing specific abnormal results, such as chest computed tomography recommended for outpatient chest radiograph abnormal results,^27^ even that yield can be low, and such data are not available for most clinical presentations. Our taxonomy provides a mechanism that will allow us to study more informed recommendation practices.

### Limitations

This study has some limitations, primarily its single-site design and retrospective nature. We did not establish or evaluate the clinical necessity of recommendations, and we do not know which recommendation or how the recommendations in each category were fulfilled. Furthermore, 1 year after the intervention, half of recommendations for additional imaging were still not complete; multifaceted quality improvement initiatives (such as feedback reporting, academic detailing, and incentives for complete language if making recommendations for additional imaging) may be needed to enhance the adoption of this intervention by radiologists.

## Conclusions

Using a newly validated taxonomy that enables analysis of radiologist recommendations for additional imaging, this cohort study has shown that the voluntary use of a closed-loop communication tool was associated with a significantly increased completeness (including time frame, modality, and reason) of recommendations for additional imaging and that a forcing function is likely required beyond free-text dictation to ensure completeness. Furthermore, ambiguous free-text recommendation language persists even when complete recommendations are filed in the closed-loop communication tool.

## Notes

Supplement 1. **eFigure.** The ARRC Closed-Loop Communication Tool Interface

Supplement 2.
Data Sharing Statement

## References

[1] Cochon LR, Kapoor N, Carrodeguas E, . Variation in follow-up imaging recommendations in radiology reports: patient, modality, and radiologist predictors. Radiology. 2019;291(3):700-707. doi:10.1148/radiol.2019182826 31063082 PMC7526331

[2] Kapoor N, Lacson R, Cochon LR, Boland GW, Khorasani R. Radiologists’ self-assessment versus peer assessment of perceived probability of recommending additional imaging. J Am Coll Radiol. 2020;17(4):504-510. doi:10.1016/j.jacr.2019.11.022 31901429

[3] Lacson R, Cochon L, Ching PR, . Integrity of clinical information in radiology reports documenting pulmonary nodules. J Am Med Inform Assoc. 2021;28(1):80-85. doi:10.1093/jamia/ocaa209 33094346 PMC7810451

[4] Bobbin MD, Ip IK, Sahni VA, Shinagare AB, Khorasani R. Focal cystic pancreatic lesion follow-up recommendations after publication of ACR white paper on managing incidental findings. J Am Coll Radiol. 2017;14(6):757-764. doi:10.1016/j.jacr.2017.01.044 28476609

[5] Nemer JS, Kazam JK, Wong TT. Trends of follow-up recommendations made on musculoskeletal MRI reports. AJR Am J Roentgenol. 2020;214(3):630-635. doi:10.2214/AJR.19.21770 31887094

[6] You JJ, Laupacis A, Newman A, Bell CM. Non-adherence to recommendations for further testing after outpatient CT and MRI. Am J Med. 2010;123(6):557.e1-557.e8. doi:10.1016/j.amjmed.2009.11.018 20569765

[7] Mabotuwana T, Hall CS, Hombal V, . Automated tracking of follow-up imaging recommendations. AJR Am J Roentgenol. 2019;212(6):1287-1294. doi:10.2214/AJR.18.20586 30860895

[8] Mabotuwana T, Hall CS, Hombal V, Dalal S, Gunn ML. Impact of follow-up imaging recommendation specificity on adherence. Stud Health Technol Inform. 2022;295:87-90. doi:10.3233/SHTI220667 35773813

[9] Kapoor N, Lynch EA, Lacson R, . Predictors of completion of clinically necessary radiologist-recommended follow-up imaging: assessment using an automated closed-loop communication and tracking tool. AJR Am J Roentgenol. 2023;1-12. doi:10.2214/AJR.22.28378 36287625 PMC12831625

[10] Kadom N, Venkatesh AK, Shugarman SA, Burleson JH, Moore CL, Seidenwurm D. Novel quality measure set: closing the completion loop on radiology follow-up recommendations for noncritical actionable incidental findings. J Am Coll Radiol. 2022;19(7):881-890. doi:10.1016/j.jacr.2022.03.017 35606263

[11] Hammer MM, Kapoor N, Desai SP, . Adoption of a closed-loop communication tool to establish and execute a collaborative follow-up plan for incidental pulmonary nodules. AJR Am J Roentgenol. 2019;212(5):1077-1081. doi:10.2214/AJR.18.20692 30779667 PMC7528936

[12] Shinagare AB, Lacson R, Boland GW, . Radiologist preferences, agreement, and variability in phrases used to convey diagnostic certainty in radiology reports. J Am Coll Radiol. 2019;16(4, pt A):458-464. doi:10.1016/j.jacr.2018.09.052 30584042

[13] Shinagare AB, Alper DP, Hashemi SR, . Early adoption of a certainty scale to improve diagnostic certainty communication. J Am Coll Radiol. 2020;17(10):1276-1284. doi:10.1016/j.jacr.2020.03.033 32387371

[14] Trinh TW, Shinagare AB, Glazer DI, . Radiology report template optimization at an academic medical center. AJR Am J Roentgenol. 2019;213(5):1008-1014. doi:10.2214/AJR.19.21451 31414884

[15] Bagga B, Fansiwala K, Thomas S, . Outcomes of incidental lung nodules with structured recommendations and electronic tracking. J Am Coll Radiol. 2022;19(3):407-414. doi:10.1016/j.jacr.2021.09.046 34896068

[16] Lewin K. Field Theory in Social Science: Selected Theoretical Papers. Harper & Brothers; 1951.

[17] Wandtke B, Gallagher S. Reducing delay in diagnosis: multistage recommendation tracking. AJR Am J Roentgenol. 2017;209(5):970-975. doi:10.2214/AJR.17.18332 28742377

[18] Mabotuwana T, Hombal V, Dalal S, Hall CS, Gunn M. Determining adherence to follow-up imaging recommendations. J Am Coll Radiol. 2018;15(3, pt A):422-428. doi:10.1016/j.jacr.2017.11.022 29502651

[19] White T, Aronson MD, Sternberg SB, . Analysis of radiology report recommendation characteristics and rate of recommended action performance. JAMA Netw Open. 2022;5(7):e2222549. doi:10.1001/jamanetworkopen.2022.22549 35867062 PMC9308057

[20] Meier FA, Zarbo RJ, Varney RC, . Amended reports: development and validation of a taxonomy of defects. Am J Clin Pathol. 2008;130(2):238-246. doi:10.1309/9UPELFVQU5WLCUHX 18628093

[21] Jacobs S, O’Beirne M, Derfiingher LP, Vlach L, Rosser W, Drummond N. Errors and adverse events in family medicine: developing and validating a Canadian taxonomy of errors. Can Fam Physician. 2007;53(2):270-276. 17872644 PMC1949126

[22] Itoh K, Omata N, Andersen HB. A human error taxonomy for analysing healthcare incident reports: assessing reporting culture and its effects on safety performance. J Risk Res. 2009;12(3-4):485-511. doi:10.1080/13669870903047513

[23] Devlin J, Chang MW, Lee K, Toutanova K. BERT: pre-training of deep bidirectional transformers for language understanding. *arXiv*. Preprint posted online May 24, 2019. doi:10.48550/arXiv.1810.04805

[24] Lacson R, Prevedello LM, Andriole KP, . Four-year impact of an alert notification system on closed-loop communication of critical test results. AJR Am J Roentgenol. 2014;203(5):933-938. doi:10.2214/AJR.14.13064 25341129 PMC4426858

[25] Lacson R, O’Connor SD, Sahni VA, . Impact of an electronic alert notification system embedded in radiologists’ workflow on closed-loop communication of critical results: a time series analysis. BMJ Qual Saf. 2016;25(7):518-524. doi:10.1136/bmjqs-2015-004276 26374896

[26] O’Connor SD, Dalal AK, Sahni VA, Lacson R, Khorasani R. Does integrating nonurgent, clinically significant radiology alerts within the electronic health record impact closed-loop communication and follow-up? J Am Med Inform Assoc. 2016;23(2):333-338. doi:10.1093/jamia/ocv105 26335982 PMC5009922

[27] Harvey HB, Gilman MD, Wu CC, . Diagnostic yield of recommendations for chest CT examination prompted by outpatient chest radiographic findings. Radiology. 2015;275(1):262-271. doi:10.1148/radiol.14140583 25531242 PMC4455667

