# Supplementary material for: Development and Assessment of an Information Technology Intervention to Improve the Clarity of Radiologist Follow-up Recommendations
Source: JAMA Netw Open. 2023 Mar 31;6(3):e236178. doi: 10.1001/jamanetworkopen.2023.6178 (PMC10066458; doi:10.1001/jamanetworkopen.2023.6178)
Supplement: Supplement 1. — eFigure. The ARRC Closed-Loop Communication Tool Interface [file jamanetwopen-e236178-s001.pdf]

Supplementary Online Content

Guenette JP, Kapoor N, Lacson R, et al. Development and assessment of an information technology intervention to improve the clarity of radiologist follow-up recommendations. *JAMA Netw Open.* 2023;6(3):e236178. doi:10.1001/jamanetworkopen.2023.6178

eFigure. The ARRC Closed-Loop Communication Tool Interface

This supplementary material has been provided by the authors to give readers additional information about their work.

© 2023 Guenette JP et al. *JAMA Network Open.*

eFigure. The ARRC Closed-Loop Communication Tool Interface

(A) The radiologist interface, which requires that the radiologist specify a discrete time interval, imaging modality, and reason for follow-up.

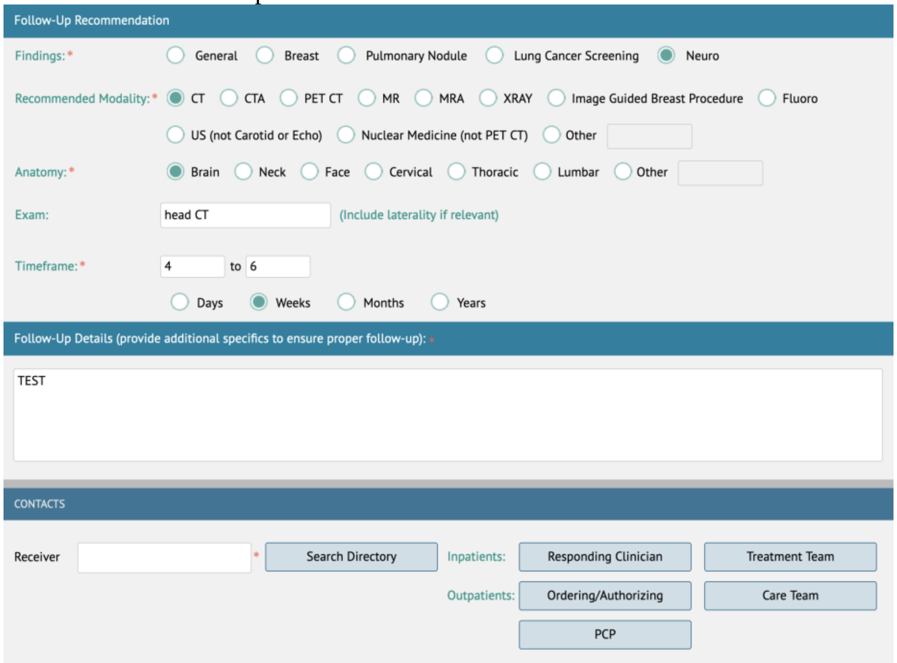

(B) The ordering provider interface, which displays the recommendation with all complete attributes and to which the ordering provider explicitly agrees, disagrees, or modifies, closing the loop.

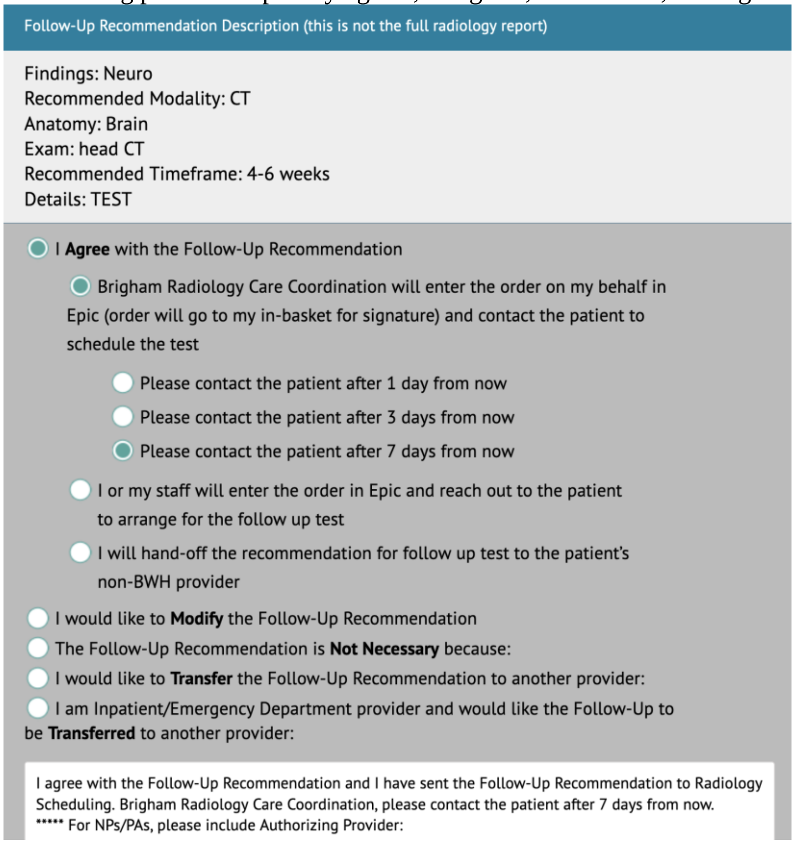

© 2023 Guenette JP et al. *JAMA Network Open.*
