# Supplementary material for: Development and Assessment of an Information Technology Intervention to Improve the Clarity of Radiologist Follow-up Recommendations
Source: JAMA Netw Open. 2023 Mar 31;6(3):e236178. doi: 10.1001/jamanetworkopen.2023.6178 (PMC10066458; doi:10.1001/jamanetworkopen.2023.6178)
Supplement: Supplement 2. — Data Sharing Statement [file jamanetwopen-e236178-s002.pdf]

Data Sharing Statement

Guenette. Development and Assessment of an Information Technology Intervention to Improve the Clarity of Radiologist Follow-up Recommendations. *JAMA Netw Open*. Published March 31, 2023. doi:10.1001/jamanetworkopen.2023.6178

Data
Data available: Yes
Data types: Deidentified participant data
How to access data: jpguenette@bwh.harvard.edu
When available: With publication

Supporting Documents
Document types: Statistical/analytic code
How to access documents: jpguenette@bwh.harvard.edu
When available: With publication

Additional Information
Who can access the data: researchers whose proposed use of the data has been approved
Types of analyses: any purpose
Mechanisms of data availability: with a signed data access agreement
